# Atractylodin Suppresses TGF-β-Mediated Epithelial-Mesenchymal Transition in Alveolar Epithelial Cells and Attenuates Bleomycin-Induced Pulmonary Fibrosis in Mice

**DOI:** 10.3390/ijms222011152

**Published:** 2021-10-15

**Authors:** Kai-Wei Chang, Xiang Zhang, Shih-Chao Lin, Yu-Chao Lin, Chia-Hsiang Li, Ivan Akhrymuk, Sheng-Hao Lin, Chi-Chien Lin

**Affiliations:** 1 Department of Chest Surgery, Tung’s Taichung MetroHarbor Hospital, Taichung 435, Taiwan; t11249@ms.sltung.com.tw; 2 Institute of Biomedical Science, National Chung-Hsing University, Taichung 402, Taiwan; 3 Department of Molecular Medicine and Surgery, Karolinska Institute, 17176 Stockholm, Sweden; xiang.zhang@ki.se; 4 Bachelor Degree Program in Marine Biotechnology, College of Life Sciences, National Taiwan Ocean University, Keelung 202, Taiwan; sclin@mail.ntou.edu.tw; 5 Graduate Institute of Biomedical Science, China Medical University, Taichung 404, Taiwan; D10001@mail.cmuh.org.tw (Y.-C.L.); d10724@mail.cmuh.org.tw (C.-H.L.); 6 School of Medicine, China Medical University, Taichung 404, Taiwan; 7 Division of Pulmonary and Critical Care Medicine, Department of Internal Medicine, China Medical University Hospital, Taichung 404, Taiwan; 8 Department of Biomedical Science and Pathobiology, Virginia-Maryland College of Veterinary Medicine, Virginia Polytechnic Institute and State University, Blacksburg, VA 24060, USA; Iakhrymu@vt.edu; 9 Division of Chest Medicine, Department of Internal Medicine, Changhua Christian Hospital, Changhua 500, Taiwan; 10 Institute of Genomics and Bioinformatics, National Chung Hsing University, Taichung 402, Taiwan; 11 Department of Recreation and Holistic Wellness, MingDao University, Changhua 523, Taiwan; 12 Program in Translational Medicine, The iEGG and Animal Biotechnology Center, National Chung-Hsing University, Taichung 402, Taiwan; 13 Department of Medical Research, China Medical University Hospital, Taichung 404, Taiwan; 14 Department of Medical Research, Taichung Veterans General Hospital, Taichung 407, Taiwan; 15 Department of Pharmacology, College of Medicine, Kaohsiung Medical University, Kaohsiung 807, Taiwan

**Keywords:** idiopathic pulmonary fibrosis, transforming growth factor-beta 1, epithelial-mesenchymal transition, atractylodin, Smad2/3, MAPK

## Abstract

Idiopathic pulmonary fibrosis (IPF) is characterized by fibrotic change in alveolar epithelial cells and leads to the irreversible deterioration of pulmonary function. Transforming growth factor-beta 1 (TGF-β1)-induced epithelial-mesenchymal transition (EMT) in type 2 lung epithelial cells contributes to excessive collagen deposition and plays an important role in IPF. Atractylodin (ATL) is a kind of herbal medicine that has been proven to protect intestinal inflammation and attenuate acute lung injury. Our study aimed to determine whether EMT played a crucial role in the pathogenesis of pulmonary fibrosis and whether EMT can be utilized as a therapeutic target by ATL treatment to mitigate IPF. To address this topic, we took two steps to investigate: 1. Utilization of anin vitro EMT model by treating alveolar epithelial cells (A549 cells) with TGF-β1 followed by ATL treatment for elucidating the underlying pathways, including Smad2/3 hyperphosphorylation, mitogen-activated protein kinase (MAPK) pathway overexpression, Snail and Slug upregulation, and loss of E-cadherin. Utilization of an in vivo lung injury model by treating bleomycin on mice followed by ATL treatment to demonstrate the therapeutic effectiveness, such as, less collagen deposition and lower E-cadherin expression. In conclusion, ATL attenuates TGF-β1-induced EMT in A549 cells and bleomycin-induced pulmonary fibrosis in mice.

## 1. Introduction

Idiopathic pulmonary fibrosis (IPF) is considered a chronic inflammatory disorder that gradually progresses to irreversible lung tissue fibrosis [1]. The cause of IPF is uncertain, and the clinical course is unpredictable. It has a poor prognosis with median survival of 2–4 years after diagnosis [2]. The major characteristics of IPF are alveolar structure damage and flourishing extracellular matrix (ECM) deposition in the basement membrane and interstitial tissue [3]. The possible mechanisms include abnormal fibroblast proliferation and transformation, myofibroblast phenoconversion, and epithelial mesenchymal transition [4,5]. Recruited fibroblasts begin to produce and deposit large amounts of ECM proteins, including collagen type I and III [6]. Myofibroblasts, α-smooth muscle actin (α-SMA)-expressing fibroblasts, even possess a greater ability to produce type I collagen than fibroblasts [7]. In 2014, the U.S. Food and Drug Administration (FDA) recognized Nintedanib and Pirfenidone for IPF treatment due to slowed decline in forced vital capacity (FVC) over 1 year, but there was no numerical trend suggesting a mortality benefit [8]. Here, we try to clarify the pathogenesis of IPF and search for natural safe and effective therapeutic drugs.

Previous studies revealed the correlation between IPF and aberrant epithelial mesenchymal transition (EMT) [9], characterized by loss of the epithelial marker E-cadherin and expression of mesenchymal markers vimentin and fibronectin [10]. Transforming growth factor-beta 1 (TGF-β1) is one of the most studied fibrogenic cytokines, controlling the development and disease progression of organ fibrosis [11], including IPF [12]. TGF-β1-induced EMT in alveolar epithelial cells is mediated through Smad-dependent or non-Smad pathways [13]. TGF-β1 mainly depends on the canonical Smad signaling pathway: TGF-β1 induces the phosphorylation of Smad2/3 to form complexes with Smad4, and translocates into the nucleus to regulate target gene expression [14]. Accumulation of nuclear Smad complexes can finally induce the expression of transcription factors (Snail, Slug, ZEB, Twist, and SIP-1) and trigger EMT [15]. On the other hand, TGF-β1 family ligands can also activate MAPK, PI3K, and RHO cascades as non-Smad signaling pathways [16,17,18]. Therefore, we assumed that EMT might be alleviated via inhibiting TGF-β1 signaling which may subsequently effectively assist the treatment of IPF.

Some natural products have been reported to have various pharmacological activities, such as antioxidant and anti-inflammatory properties [19]. Atractylodin (ATL), a polyethylene alkyne extracted from Atractylodis rhizoma, is a traditional herbal medicine widely used in Korea for gastritis and gastric ulcers [20]. It has been reported to ameliorate intestinal inflammation via inhibiting both pro-inflammatory cytokines (TNF-α, IL-1β, and IL-6) and inflammatory mediators (iNOS and NF-κB) [21]. It also attenuates lipopolysaccharide-induced acute lung injury by suppressing activation of TLR4-NF-κB and -MAPK pathway and the NLRP3 inflammasome [22]. However, the effect of ATL on pulmonary fibrosis has not been previously reported. In this study, we propose the safe dosage of atractylodin and confirm the anti-EMT pathway via inhibiting TGF-β1/Smad and MAPK signaling cascades in human alveolar epithelial A549 cells and in mice.

## 2. Results

### 2.1. Effect of Atractylodin on Cell Viability of TGF-β1-Induced A549 Cells

**Figure 1:**
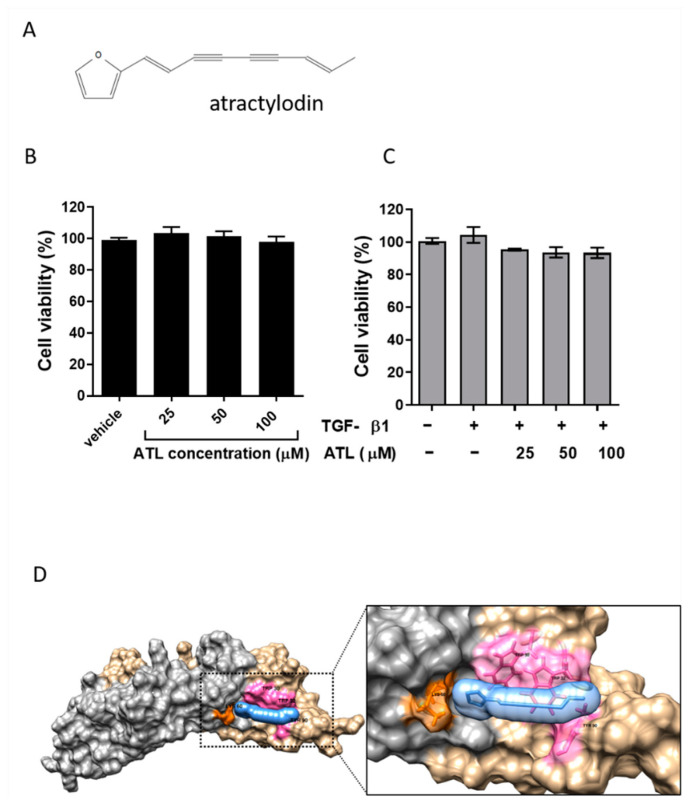
Atractylodin caused neglectable cytotoxicity on A549 cells with or without TGF-β1. (**A**) The molecular structure of atractylodin. (**B**) The cell viability of A549 cells treated with ATL (0, 25, 50, 100 μM) for 24 h was determined by MTT assay. (**C**) The cell viability of A549 cells treated with different concentrations of ATL in the presence of TGFβ1 (2 ng/mL) was measured by MTT assay. Values represent the mean ± SEM from triplicate samples for each treatment. (**D**) 3D structure of the docking model. Atractylodin is indicated in blue; the homodimer is composed of TGF-β1A (in tan) and TGF-β1B (in grey); amino acid residuals interacting with TGF-β1 receptor are labeled in pink for Trp30, Trp32, Tyr90 of TGF1-β1A and in orange for Lys60 of TGF-β1B. Values represent the mean ± SEM from triplicate samples for each treatment.

To rule out the possibility of atractylodin per se might induce cytotoxicity and thus interfere the experiments afterwards, we treated A549 cells with various concentrations of atractylodin between 0 and 100 μM. As shown in Figure 1A, atractylodin had no toxic effect on A549 cells even at the concentration of 100 μM for 24 h in MTT assays. In Figure 1B, there was also no decline in cell viability in the presence of TGF-β1 (2 ng/mL). In accordance with the cellular assays, the results from the computational docking implied that atractylodin could strongly interact with homodimer of TGF-β1 via a pi-alkyl interaction (Figure 1D, Supplemental Figure S1). These results indicate that atractylodin and TGF-β1 induced no cytotoxicity within the concentrations we tested and that atractylodin could potentially interact with TGF-β1 to intervene with the physiological functions of TGF-β1, which we further validate in the later experiments.

### 2.2. Atractylodin Alleviates TGF-β1-Induced EMT in A549 Cells

**Figure 2:**
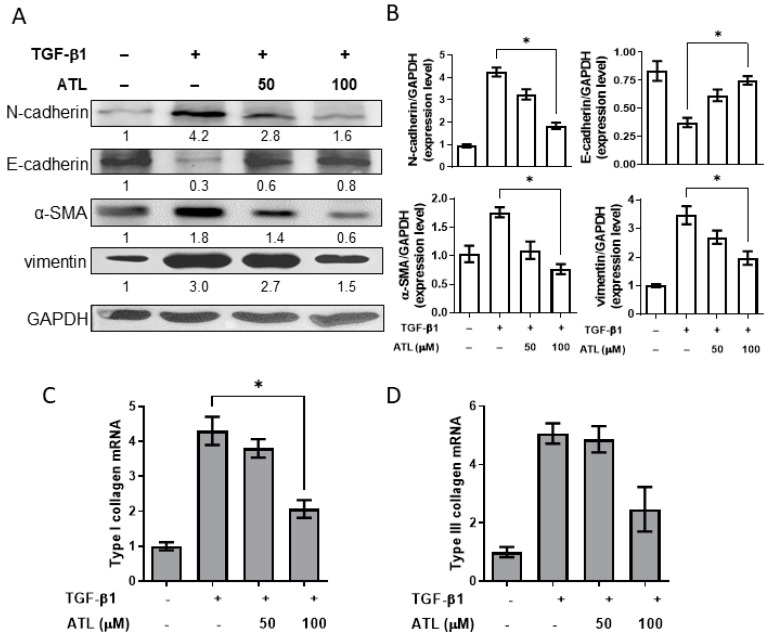
Effect of atractylodin stymied on TGF-β1-induced EMT-associated protein expressions in A549 cells. A549 cells were pretreated with ATL for 1 h followed by TGF-β1 (2 ng/mL) stimulation for an additional 24 h. Cells treated with DMSO were set up as the control groups. (**A**) Protein expression levels of N-cadherin, E-cadherin, α-SMA, and vimentin were measured by Western blot assay. (**B**) Quantitation of Western blot signal intensities by ImageJ software. (**C**) The transcriptional expressions of type I collagen and (**D**) type III collagen were conducted by RT-qPCR. Values represent the mean ± SEM from triplicate samples for each treatment. (*) *p* < 0.05 versus TGF-β1 + 0.1% DMSO-treated control, as determined by non-parametric Kruskal–Wallis test and all pairwise multiple comparison procedures (Dunn’s Method).

Since TGF-β1 has a key role in EMT for fibrosis [15], we referred the previous report to establish an in vitro EMT model of TGF-β1-treated A549 cells to evaluate the effects of ATL on EMT change [23]. As illustrated in Figure 2A, decreased protein expression of E-cadherin (epithelial phenotype) and increased expression of α-SMA and vimentin (mesenchymal phenotype) were found by treating cells with TGF-β1. A549 cells were further exposed to 100 μM atractylodin in the presence of TGF-β1, and E-cadherin up-regulation, as well as α-SMA and vimentin down-regulation, were identified compared to the group with TGF-β1 alone. Quantitative Western blot analysis is illustrated in Figure 2B, where these quantitative data were consolidated with the transcriptional expression of type I and III collagen. The mRNA levels of type I and III collagen in TGF-β1-treated cells steeply increased, and 100 μM atractylodin considerably decreased the mRNA levels of type I in a dose-dependent manner (Figure 2C,D).

### 2.3. Atractylodin Inhibits EMT-Related Transcription Factor Expression in A549 Cells

**Figure 3:**
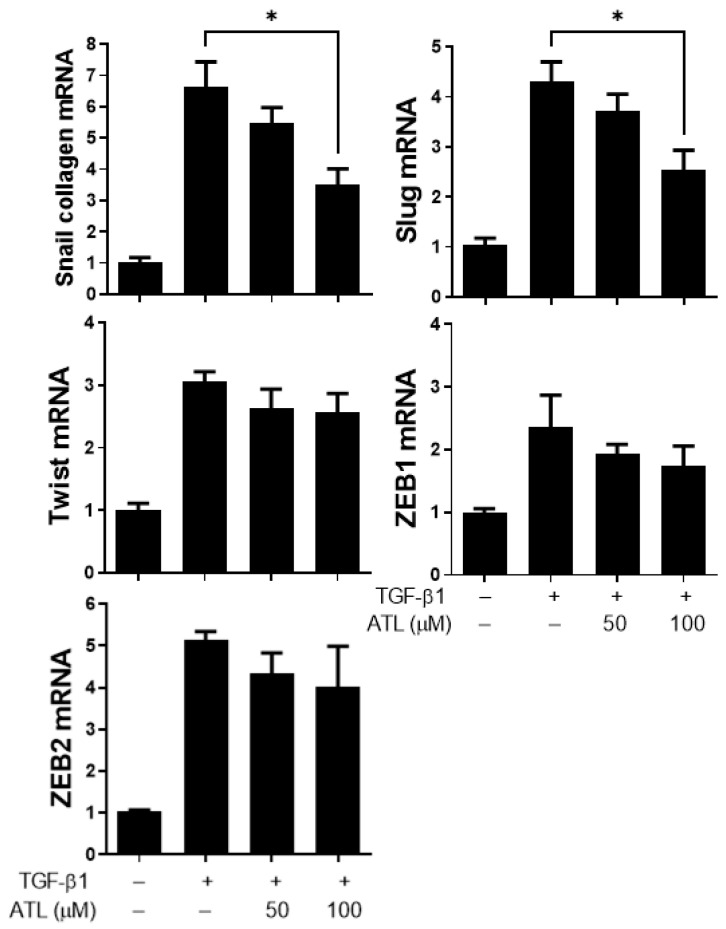
Atractylodin moderately reduced EMT-related transcription factor expression in TGF-β1-treated A549 cells. A549 cells were pretreated with ATL for 1 h followed by TGF-β1 (2 ng/mL) stimulation for an additional 24 h. Cells treated with DMSO were set up as the control group. Real-time PCR was exploited for quantifying the expressional changes of EMT-related transcription factors, including Snail, Slug, Twist, ZEB1, and ZEB2. Values represent the mean ± SEM from triplicate samples for each treatment. (*) *p* < 0.05 versus TGF-β1 + 0.1% DMSO-treated control, as determined by non-parametric Kruskal–Wallis test and all pairwise multiple comparison procedures (Dunn’s Method).

Many EMT promoting transcription factors, such as Snail [24,25], Slug [26,27], Twist [28], ZEB1 [29], and ZEB2 [30], are induced by TGF-β1 and have been proven to suppress E-cadherin expression [26,27]. To determine the mechanisms by which atractylodin could inhibit EMT by increasing E-cadherin in TGF- β1-induced A549 cells, we used RT-qPCR to measure the expression levels of these associated transcription factors. The results reported that the mRNA expression levels of Snail, Slug, Twist, ZEB1, and ZEB2 were significantly increased in TGF-β1-stimulated cells (Figure 3). Treatment with 100 µM atractylodin dramatically inhibited the expression of Snail and Slug mRNA (Figure 3); however, there was no obvious difference in the expression of Twist, ZEB1 and ZEB2 mRNA (Figure 3). These results suggest that Snail and Slug down-regulation might be the key mechanism by which atractylodin increases E-cadherin in TGF- β1-induced A549 cells.

### 2.4. Atractylodin Reduces Smad-Dependent Pathway Activation in A549 Cells

**Figure 4:**
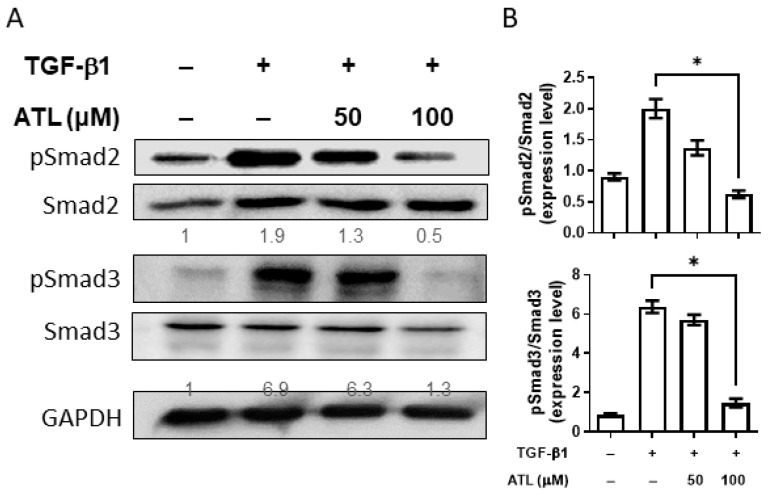
Atractylodin suppressed Smad-dependent pathway activation triggered by TGF-β1 in A549 cells. A549 cells were pretreated with ATL for 1 h followed by TGF-β1 (2 ng/mL) stimulation for an additional 6 h. Cells treated with DMSO were set up as the control group. (**A**) Protein expression levels of p-Smad2, p-Smad3, Smad2, and Smad3 were measured by Western blot assay. (**B**) Quantitation of Western blot signal intensities with ImageJ software. Values represent the mean ± SEM from triplicate samples for each treatment. (*) *p* < 0.05 versus TGF-β1 + 0.1% DMSO-treated control, as determined by non-parametric Kruskal–Wallis test and all pairwise multiple comparison procedures (Dunn’s Method).

From previous studies, we know that the expression of Snail and Slug are regulated by Smad-dependent or Smad-independent signaling pathways [15]. To determine the mechanisms, we first investigated the effects of atractylodin on Smad pathway activation. After A549 cells were exposed to TGF-β1, the levels of phosphorylated Smad2 and Smad3 were significantly increased (Figure 4A). Additionally, we found atractylodin markedly inhibited the phosphorylation of both Smad2 and Smad3, especially at the concentration of 100 μM. The ratio of p-Smad2/Smad2 and p-Smad3/Smad3 between different groups were compared after quantification of Western blot signals (Figure 4B). These findings suggest that atractylodin may inhibit the expression of Snail and Slug through the Smad-dependent pathway.

### 2.5. Atractylodin Suppresses Smad-Independent Pathway Activation in A549 Cells

**Figure 5:**
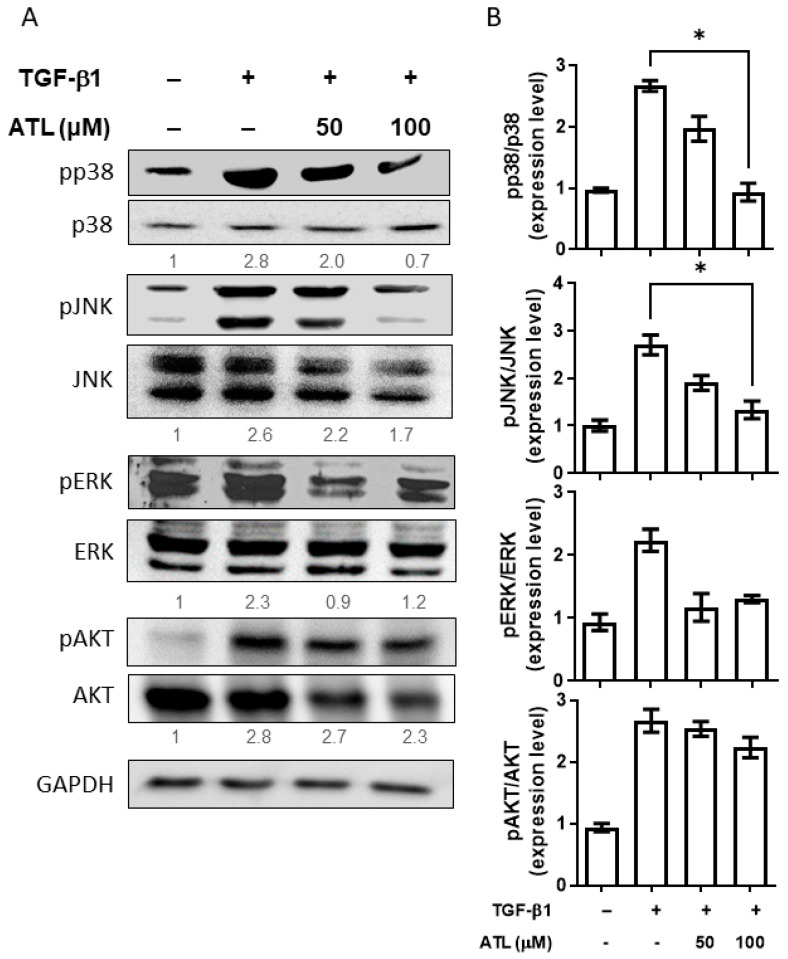
Atractylodin reduced Smad-independent pathway activated by TGF-β1 in A549 cells. A549 cells were pretreated with ATL for 1 h followed by TGF-β1 (2 ng/mL) stimulation for an additional 6 h. Cells treated with DMSO were set up as the control group. (**A**) Protein expression levels of phospho- and non-phospho- p38, JNK, ERK, and AKT were measured by Western blot assay. (**B**) Quantitation of Western blot signal intensities by ImageJ software. Values represent the mean ± SEM from triplicate samples for each treatment. (*) *p* < 0.05 versus TGF-β1 + 0.1% DMSO-treated control, as determined by non-parametric Kruskal–Wallis test and all pairwise multiple comparison procedures (Dunn’s Method).

Next, we wanted to clarify the effect of atractylodin on the Smad-independent pathway, including the MAPK and PI3K/AKT cascades [18]. Initially, A549 cells were stimulated by TGF-β1, and, using Western blot analysis, the levels of phosphorylated p38, JNK, ERK, and AKT were significantly increased (Figure 5A). After atractylodin treatment, there was an obvious decrease in the phosphorylation of p38 and JNK, implying that atractylodin might also suppress the expression of Snail and Slug in the Smad-independent pathway. Quantification of Western blot band intensity is demonstrated in Figure 5B.

### 2.6. Atractylodin Decreases BLM-Induced Pulmonary Fibrosis in Mice

**Figure 6:**
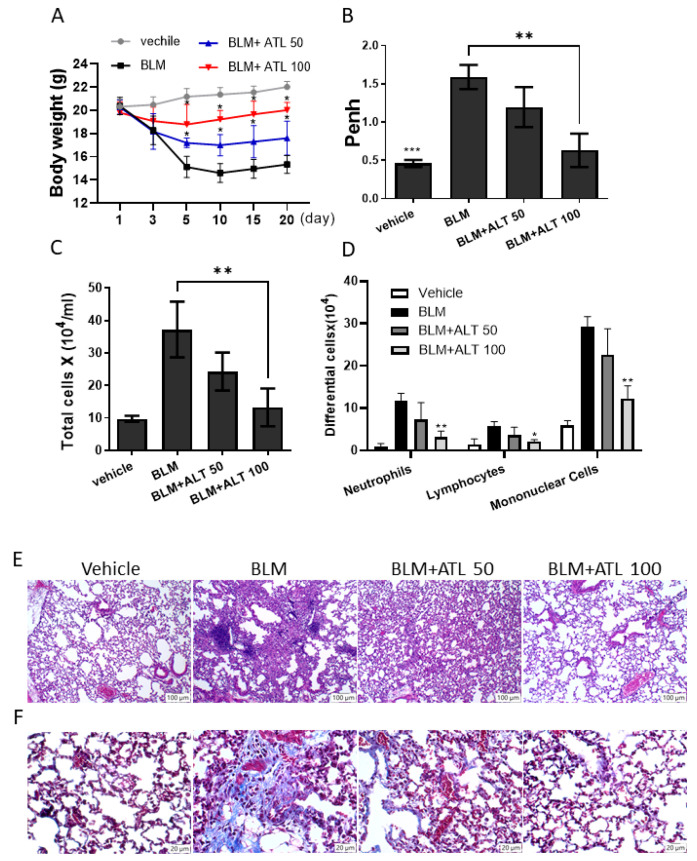
Atractylodin ameliorated BLM-induced pulmonary fibrosis. (**A**) The body weight changes in BLM-treated mice received ATL treatment 0, 50, and 100 mg/kg. (**B**) The lung function test for Penh value was performed by plethysmograph on day 21. (**C**) Numbers of total inflammatory cells and (**D**) immune cells of neutrophils, lymphocytes as well as mononuclear cells in BALF were stained with Wright-Giemsa stain and counted under the microscopy. Data are expressed as mean ± SEM of five mice in each group. (*) *p* < 0.05, (**) *p* < 0.01, (***) *p* < 0.001 versus vehicle-treated BLM model group (as control group), as determined by non-parametric Kruskal–Wallis test and all pairwise multiple comparison procedures (Dunn’s Method). (**E**) Pulmonary pathological changes in tissues from BLM treated mice with or without ATL treatments were examined followed by H&E staining (original magnifications, 400×). (**F**) Collagen deposition in lung tissues was visualized and examined by Masson’s trichrome staining (original magnifications, 400×).

To examine the effect of atractylodin in vivo, we treated mice with intratracheal instillation of bleomycin daily for 20 consecutive days. When comparing the control group and BLM-induced pulmonary fibrosis model group, we found that BLM could lead to overt body weight loss in the first 10 days, and atractylodin could reverse the body weight change to some extent (Figure 6A). Next, we evaluated the extent of pulmonary fibrosis of the mice by using the value of Penh, an indicator for lung function and airway resistance. Baseline Penh values were significantly higher in the BLM-treated model group than in the vehicle control group (Figure 6B). ATL significantly reduced airway resistance, an indicator for pulmonary fibrosis, with 100 mg/kg ATL having a better effect than 50 mg/kg ATL.

In the next step, we collected bronchial alveolar lavage fluid to compare inflammatory cells across these groups. As shown in Figure 6C, the total cell number in the BLM-treated group was much higher than the control group, and this was reversed after treatment with ATL. The situation was the same when we examined the differential counts of neutrophils, lymphocytes, and mononuclear cells separately (Figure 6D). We further explored the effect of atractylodin on pathological changes in pulmonary fibrosis of mice lung tissues by H&E staining (Figure 6E). In the control group, the structures of bronchi and alveoli were complete and clear, with eumorphic epithelial cells and scanty inflammatory cell infiltration in the lung tissues. In the BLM-treated model group, the alveolar structure was destroyed, the cytoplasm was loose and vacuolated, and the surrounding parenchyma was infiltrated by large amounts of inflammatory cells. Notably, these pathological phenomena were alleviated by supplementation with atractylodin.

Masson’s trichrome staining was further used to identify collagen deposition in the lung tissues (Figure 6F). In the mice of the control group, staining clearly showed that alveolar structure was complete with no obvious fibrous hyperplasia. On the contrary, abundant blue matrix collagen fibers were deposited in the bronchi, around the vascular wall, and in the interstitium of lung tissue in the model group, indicating that bleomycin-induced pulmonary fibrosis in mice was significantly increased. Compared with the model group, the intervention of atractylodin noticeably attenuated collagen deposition and normalized alveolar structure. These results indicate that atractylodin delayed the progression of lung fibrosis by reducing collagen deposition.

### 2.7. Atractylodin Down-Regulates BLM-Induced EMT in Mice Lung Tissues

**Figure 7:**
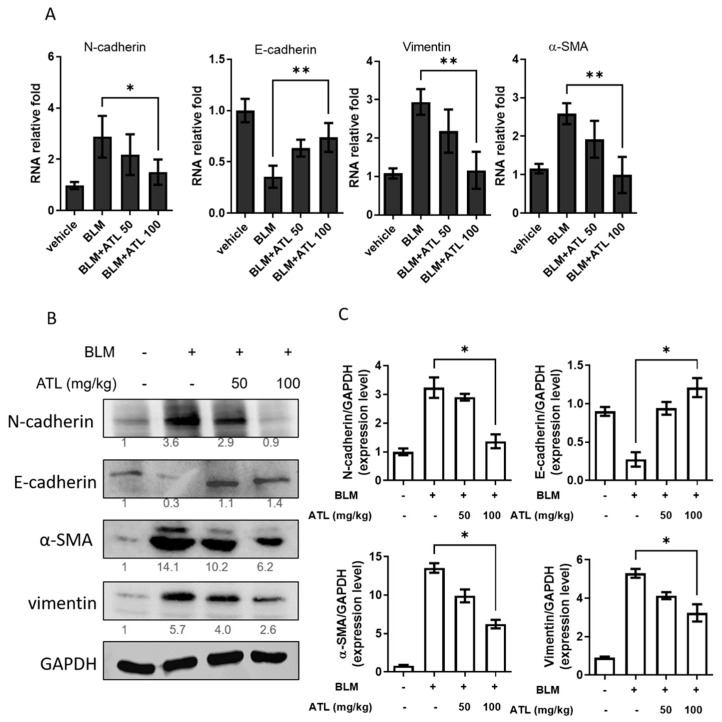
Atractylodin reduced BLM-induced EMT in mice pulmonary tissues. Lung tissue homogenates were collected on day 21 from each group of mice. (**A**) Relative mRNA expression levels of E-cadherin, α-SMA, and vimentin were measured with real-time PCR. (**B**) Protein expression levels of N-cadherin, E-cadherin, α-SMA, and vimentin were assessed with Western blot assay. (**C**) Quantitation of Western blot signal intensities by ImageJ software. Data are expressed as mean ± SEM of five mice in each group. (*) *p* < 0.05, and (**) *p* < 0.01 versus vehicle-treated BLM model group (as control group), as determined by non-parametric Kruskal–Wallis test and all pairwise multiple comparison procedures (Dunn’s Method).

To determine if BLM treatment would induce EMT in the lungs, E-cadherin expression levels were measured by qRT-PCR and Western blot analysis. We found a significant down-regulation of both E-cadherin mRNA and protein expression in BLM-induced pulmonary tissues compared with the control group (Figure 7A,B). Notably, 100 mg/kg atractylodin treatment reversed the increasing expression of N-cadherin and decreasing expression of E-cadherin in BLM stimulation. In addition, the mRNA and protein expression of mesenchymal markers, α-SMA and vimentin, were also elevated by BLM and suppressed by 100 mg/kg atractylodin. The quantitation of Western blot signal intensities of these biomarkers is shown in Figure 7C. These results represent the therapeutic effects of atractylodin with BLM-induced EMT in mice pulmonary tissues.

## 3. Discussion

Fibrotic change after alveolar epithelial cell injury is the major factor leading to the irreversible deterioration in IPF patients [31]. Therefore, all possible anti-fibrotic mechanisms were investigated to advance IPF therapy [32]. During the process of pulmonary fibrosis, activated type II alveolar epithelial cells undergo EMT and transform into fibroblasts and myofibroblasts [9]. In an experimental fibrosis model, approximately one-third of the lung fibroblasts derived from the lung epithelial cells underwent EMT 2 weeks after bleomycin administration [33]. In this study, we sought to confirm that atractylodin possesses effective therapeutic effects to lower pulmonary fibrotic change via alleviating TGF-β1-mediated epithelial-mesenchymal transition.

Transforming growth factor-beta 1 (TGF-β1), a fibrogenic cytokine, can stimulate EMT in pulmonary fibrosis [13]. The in vitro model that epithelial to mesenchymal transition is triggered by TGF-β1 in A549 cells and in vivo BLM-treated in mouse pulmonary tissues was evaluated for ATL effects by observing the expression levels of EMT-related biomarkers. Our results confirmed that TGF-β1 decreased the expression of the epithelial-specific biomarker E-cadherin and elevated expression levels of mesenchymal biomarkers N-cadherin, α-SMA, and vimentin and indicated that, ATL can attenuate such decreasing shifts, suggesting that ATL might reverse the undesired changes of EMT. We also noted that ATL reduced type I collagen transcript production (Figure 2), suggesting that this reversed effects by ATL appeared to involve transcriptional and translational steps.

The most critical hallmark of EMT is the downregulation of E-cadherin [34], which is mediated by its transcriptional repression through the binding of EMT-activating transcription factors (Snail, Slug, ZEB, Twist, and SIP-1) to E-boxes present in the E-cadherin promoter [35,36]. In our study, ATL 100 μM was found to repress the mRNA expression of both Snail and Slug, implying ATL suppressed EMT by down-regulation of Snail and Slug transcription factors.

The EMT processes induced by TGF-β1 can be classified into Smad-dependent and Smad-independent pathways [13]. In the canonical Smad-dependent pathway, TGF-β1 binds to the type II (TβRII) and type I (TβRI) receptors on the cell membrane, where TβRII phosphorylates TβRI. This induces the phosphorylation of Smad2 and Smad3, forming a heterocomplex with the Co-Smad Smad4, and finally translocates into the nucleus to cooperate with DNA-binding transcription factors to regulate the expression of transcription factors (Snail, Slug, ZEB, Twist, and SIP-1) [37]. In A549 cells exposed to TGF-β1, phosphorylated Smad2 and Smad3 were significantly increased. ATL treatment reduced the level of TGF-β1-stimulated Smad2 and Smad3 phosphorylation, indicating the capacity of ATL to inhibit the TGF-β1/Smad-dependent signaling pathway. As for the Smad-independent pathway, TGF-β1 activates the MAPK, PI3K/AKT, and RHO/ROCK cascades to regulate the expression of transcription factors related to EMT in a manner independent of Smad, resulting in the loss of E-cadherin [18,26]. Our present study shows that ATL inhibits the activation of P38, JNK, and ERK, suggesting that ATL might also down-regulate Snail and Slug via a Smad-independent pathway.

In regards of any other potential targets/pathways that might be recognized or exploited by ATL, heat shock protein (Hsp) family could be the next candidate, which is responsible for correctly folding and stabilizing proteins [38]. Increasing evidence has implied the correlation of Hsp90 and IPF by presenting the evidence of up-regulation of Hsp90 expression and activation of its ATPase function, facilitating matrix metalloproteinase activity, integrin expression, and epithelial-to-mesenchymal transition, all of which are involved in promoting the progress of fibrosis [39,40,41,42]. Additionally, Hsp90 facilitated the protein stability and folding of TGF-β receptors (TβRI and TβRII) and Src kinase, associated with the pro-fibrosis [43]. Despite lack of direct evidence in this study, we speculate that Hsp90 could be a potential target for suppression of IPF and one of the underlying mechanisms for ATL. What we found here was that ATL profoundly reduced the TGF-β-induced EMT accumulation (Figure 2) but whether Hsp90 inhibition can achieve similar results or whether Hsp90 can be affected by ATL require further investigation.

Previously, researchers focused on the protective effects of ATL on acute lung injury induced by lipopolysaccharide through Toll-like receptor 4 pathway, demonstrating that ATL is capable of harmonizing the inflammatory status via inhibiting IL-6, TNF-α, and IL-1β cytokine productions toward Th1 response [22]. Our previous work has further indicated that ATL could shift the Th1 to Th2 immunity and modulate dendritic cell functions [44]. This current study literally puts one more piece of the puzzle into the whole picture regarding the benefits and effectiveness of ATL on both acute and chronic pulmonary diseases, although detailed mechanisms of action desperately require to be elucidated.

## 4. Material and Methods

### 4.1. Cell Viability Assay

Cell viability was determined with the 3-(4,5-dimethylthiazol-2-yl)-2,5-diphenyltetrazolium bromide (MTT, Sigma-Aldrich, St. Louis, MO, USA) assay. Briefly, A549 cells (adenocarcinomic human lung alveolar type II epithelial cells) were seeded at 1 × 10^5^ cells/mL per well in 96-well plates and cultured overnight at 37 °C. Cells were pre-treated with ATL (0–100 uM) for 2 h and then subjected to treatment in the absence or presence of TGF-β1 (2 ng/mL, Sigma-Aldrich St. Louis, MO, USA, cat NO. T7039) for 24 h. The vehicle control group was treated with 0.1% DMSO solvent (Sigma-Aldrich, St. Louis, MO, USA). Following cell treatment, the cells were incubated with 10 µL of 500 µg/mL MTT solution for 4 h at 37 °C. The formazan crystals were dissolved in 150 µL of DMSO, and the absorbance was measured at 570 nm using a microplate reader (TECAN, Durham, NC, USA). The percentage of cell viability is calculated using the following formula:

Cell viability (%) = * 100 %

### 4.2. Western Blot Assay

A549 cells and lung tissues were lysed on ice in RIPA buffer containing 1% protease inhibitor cocktail (Sigma-Aldrich, St. Louis, MO, USA) and phosphatase inhibitor (Roche, Mannheim, Germany). The bicinchoninic acid assay (Cat# BC03-500, Visual protein, Taipei City, Taiwan) was used for protein quantification. Subsequently, an equal protein content (40 µg) from each sample was separated by 10% SDS–PAGE and transferred onto a polyvinylidene difluoride (PVDF) membrane (Millipore, Billerica, MA, USA). The membranes were blocked with BlockPRO™ Protein-Free Blocking Buffer for 1.5 h at room temperature, and then incubated with specific primary antibodies, E-cadherin (clone EP700Y, 1:1000, Epitomics, Burlingame, CA, USA), α-SMA (Cat#ab5694, 1:1000, Abcam, Cambridge, MA, USA), vimentin (Cat# GTX100619, 1:1000, GeneTex Inc, Texas, USA), p-p38 (clone 3D7, 1:1000, Cell Signaling Technology, Danvers, MA, USA), p38 (clone D13EE1, 1:1000, Cell Signaling Technology, Danvers, MA, USA), p-JNK (clone 81E11, 1:1000, Cell Signaling Technology, Danvers, MA, USA), JNK (Cat# 9252, 1:1000, Cell Signaling Technology, Danvers, MA, USA), p-ERK (clone E-4, 1:1000, Santa Cruz Biotechnology, CA, USA), ERK (clone H-72, 1:1000, Santa Cruz Biotechnology, CA, USA), p-AKT (Ser473) (clone D9E, 1:1000, Cell Signaling Technology, Danvers, MA, USA), AKT (clone C67E7, 1:1000, Cell Signaling Technology, Danvers, MA, USA), p-SMAD2 (Ser465/467) (clone 138D4, 1:1000, Cell Signaling Technology, Danvers, MA, USA), SMAD2 (clone D43B4, 1:1000, Cell Signaling Technology, Danvers, MA, USA), p-SMAD3 (Ser465/Ser467) (clone E8F3R, 1:1000, Cell Signaling Technology, Danvers, MA, USA), SMAD3 (clone C67H9, 1:1000, Cell Signaling Technology, Danvers, MA, USA), and GAPDH (Cat# ab8245, 1:5000, Abcam, Cambridge, MA, USA), at 4 °C. After incubation overnight, the membranes were washed with TBS-T, and then incubated with the horseradish peroxidase (HRP)-conjugated corresponding secondary antibody (Jackson ImmunoResearch Laboratories, West Grove, PA, USA) (1:10,000 dilution) for 1 h at room temperature. Immunoblots were detected using a LumiFlash™ Ultima Chemiluminescent substrate, HRP system (Visual protein, Taipei City, Taiwan; LF08–500) and visualized using a Hansor Luminescence Image System (Taichung, Taiwan). The band intensity was quantified with the ImageJ 1.47 program from the National Institutes of Health (NIH) (Bethesda, MD, USA).

### 4.3. Quantitative Real-Time PCR

Total RNA was purified from A549 cells or pulmonary tissues using TRIzol^®^ reagent (Invitrogen; Thermo Fisher Scientific, Inc., Carlsbad, CA, USA). Total of 2 µg total RNA was reverse transcribed with the use of Moloney murine leukemia virus (M-MLV) reverse transcriptase, 5X reaction buffer, dNTPs and oligo (dT) 15 primers (Promega Corporation, Madison, WI, USA). To detect the gene expression of type I collagen, type III collagen, Snail, Slug, Twist, ZEB1 and ZEB2, E-cadherin (mouse), vimentin (mouse), and α-SMA (mouse), the following primers were used as shown in supplemental Table S1. Then, quantitative real-time PCR was performed using StepOne^TM^ Real-Time PCR System (Applied Biosystems, Foster City, CA, USA). The gene expression level in samples was normalized against GAPDH, and the expression level was calculated using the delta delta threshold cycle (2^−ΔΔCt^) method.

### 4.4. Animal Care and Experimental Procedures

C57B/6 male mice (6 weeks old; 20 ± 3 g) were purchased from the National Laboratory Animal Center (Taipei, Taiwan). The animals were reared under conventional conditions: temperature-controlled room (22 ± 2 °C) with 70% humidity under a 12 h light/dark cycle and were allowed free access to food and water at the specific pathogen-free facility of National Chung Hsing University (Taichung, Taiwan). All procedures using animals were reviewed and approved (9 August 2021) by the Institutional Animal Care and Use Committee (approval number: IACUC 110-065) of National Chung Hsing University (NCHU), and the study protocols were approved by the Committee on Animal Research and Care in NCHU. The mice were randomly assigned to four experimental groups (n = 5): (1) control group, (2) bleomycin (BLM) pulmonary fibrosis model group (BLM + vehicle), (3) BLM + ATL 50 mg/kg group, (4) BLM + ATL 100 mg/kg group. To establish a pulmonary fibrosis (PF) model, the mice were given BLM (2 mg/kg, volume 20 μL/20 g body weight) dissolved in sterile PBS by intratracheal instillation. Mice receiving PBS without BLM were included as a control group. Mice received ATL (50 or 100 mg/kg per day through intraperitoneal injection) starting from one day after intratracheal instillation of BLM, for 20 consecutive days. The concentration of ATL was decided by referencing a previous report [22,45]. On day 21, mice were sacrificed with 50% CO_2_ until they were unconscious and experienced cardiac arrest. The left lung was ligated, and the right lung was gently washed three times with 0.5 mL PBS. The total number of leukocytes in the collected bronchoalveolar lavage fluid (BALF) was counted. The total cells in BALF were centrifuged and stained with Wright-Giemsa stain. We quantified the total number of each cell type on the slides by counting a total of 200 cells/slide. The bottom of the left lower lobe was fixed in 10% formalin for histological examination. The lung tissues were collected and stored at −80 °C for further experiments.

### 4.5. Measurement of Airway Hyperresponsiveness

Airway hyperresponsiveness was measured at day 20 by using the Buxco FinePointe (Buxco Electronics, Troy, NY, USA) to observe mice responding to increasing amounts of aerosolized methacholine-induced airflow obstruction in conscious unrestrained mice placed in whole-body plethysmography (Buxco, Sharon, CT, USA). Pulmonary resistance was evaluated and expressed as an enhanced pause (Penh). Mice were challenged with methacholine aerosol in increasing concentrations from 12.5 to 50 mg/mL in PBS with an ultrasonic nebulizer. Data on lung resistance were continuously collected, and mean values were selected to express changes in airway function.

### 4.6. Histopathological and Immunohistochemical Examination

On day 21, the mice were sacrificed, and lung tissues were collected and fixed in 10% formalin and then embedded in paraffin. Each section (5 μm) of tissue was stained with hematoxylin and eosin (H&E) for microscopic evaluation of peribronchial cell counts and the severity of the infiltration of inflammatory cells. Masson’s trichrome staining (Abcam, Cambridge, UK) was used to detect collagen fiber deposition in lung tissues following the manufacturer’s instructions. The collagen fibers were stained blue, the nuclei were stained black, and the background was stained red.

### 4.7. Molecular Docking

The structure of TGF-β1 homodimer was cleaved from PDB:3KFD (doi:10.1074/jbc.M109.079921), an interaction model for TGF-β1 homodimer and TGF-β receptor (type I and type II). Molecular docking was performed between the structure of atractylodin (PubChem CID: 5321047) and TGF-β1 homodimer via Autodock Vina (doi:10.1002/jcc.21334). The docking model was visualized by UCSF Chimera 1.15 (https://doi.org/10.1002/jcc.20084). The demonstration of interactions among ligands and nearby amino acid residuals was achieved by Discovery studio visualizer version v21.1.0.20298 (BIOVIA, San Diego, CA, USA).

### 4.8. Statistical Analysis

These are presented as the mean ± standard error of the mean (SEM). Each experiment was carried out at least three times independently. The statistical significance of differences between the groups were investigated using non-parametric Kruskal–Wallis test and all pairwise multiple comparison procedures (Dunn’s Method) with GraphPad Prism v9.0 software (GraphPad Software, Inc., San Diego, CA, USA). *p* < 0.05 was considered to indicate a statistically significant difference.

## 5. Conclusions

In vivo and in vitro experiments confirmed that atractylodin influenced TGF-β1-mediated EMT by down-regulating the expression of the transcription factors Snail and Slug via both Smad and non-Smad pathways. The results provide a rationale for exploiting the atractylodin’s inhibitory activity on excessive collagen deposition in lung cells and a possibility that ATL may serve as a tool for those who dedicate to investigate the EMT-related pathogenesis in various diseases, such as IPF in the further.

## Data Availability

The data that support the findings of this study are available from the corresponding author upon reasonable request.

## Supplementary Materials

The following are available online at https://www.mdpi.com/article/10.3390/ijms222011152/s1.
