# Supplementary material for: Atractylodin Suppresses TGF-β-Mediated Epithelial-Mesenchymal Transition in Alveolar Epithelial Cells and Attenuates Bleomycin-Induced Pulmonary Fibrosis in Mice
Source: Int J Mol Sci. 2021 Oct 15;22(20):11152. doi: 10.3390/ijms222011152 (PMC8570326; doi:10.3390/ijms222011152)
Supplement: Supplementary file 1 [file ijms-22-11152-s001.zip › ijms-1398618-supplementary.pdf]

Supplementary Figure S1

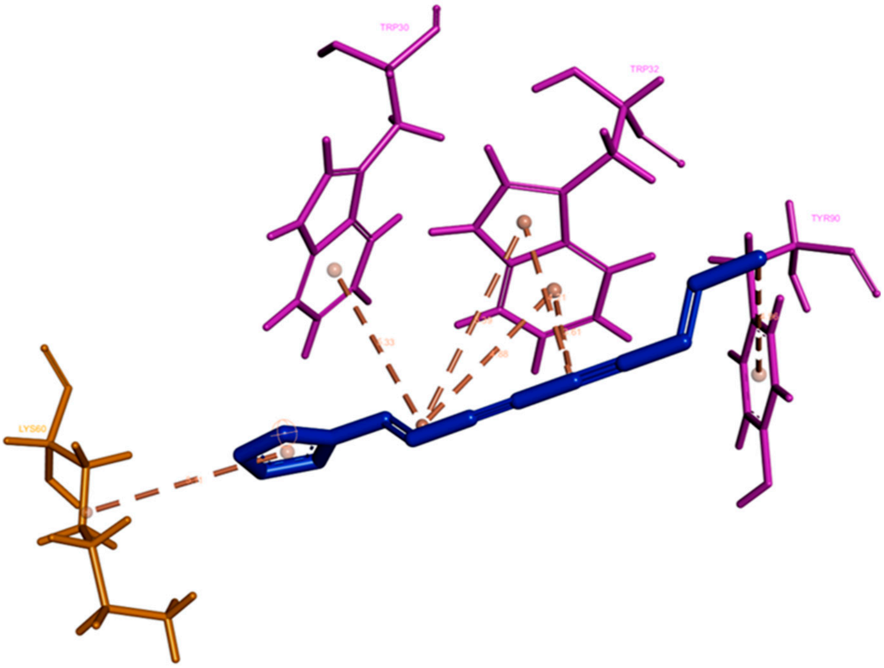

Supplemental Figure S1. Molecular interaction between atractylodin and the nearby amino acid residuals of TGF-β1. The interactions between the 4 residuals (Trp30, Trp32, Tyr90 and Lys60) with atractylodin are a pi-alkyl interaction.

Supplementary Table S1. List of primers used for real-time PCR analysis

Gene	Forward primer	Reverse primer
type I collagen	5'-CTGGCCTCGGAGGAAACTTT-3'	5'-TCCGGTTGATTTCTCATCATAGC-3'
type III collagen	5'-TGGAGTGTCTGGACCAAAAGG-3'	5'-TGATCCCAGCAATCCCAAGT-3'
SNAIL	5'- CCCCAATCGGAAGCCTAACT-3'	5'GCTGGAAGGTAAACTCTGGATTAGA-3'
SLUG	5'- TGCGGCAAGGCGTTTT -3'	5'-TCCCCCGTGTGAGTTCTAATG-3'
TWIST	5'-ACGCTGCCCTCGGACAA-3'	5'- TCGCTCTGGAGGACCTGGTA-3'
ZEB1	5'-CACCATCCCCATCACCTCTAA-3'	5'-GCACCCTCAGCTGTGTACAAGT-3'
ZEB2	5'-GGAGAAAGTACCAGCGGAAACA-3'	5'-TCTAGGCCTGACATGTAGTCTTGTG-3'
E-cadherin	5'-GTCACTGACACCAACGATAATCCT-3	5'-GGCACCTGACCCTTGTACGT-3'
vimentin	5'-AATGACCGCTTCGCCAACT-3’	5'-ATCTTATTCTGCTGCTCCAGGAA-3'
α-SMA	5'-TCCCTGGAGAAGAGCTACGAACT-3'	5'-AAGCGTTCGTTTCCAATGGT-3'
GAPDH	5'-ACCCAGAAGACTGTGGATGG-3'	5'-TGCTGTAGCCAAATTCGTTG-3'
